# Genome-wide analysis of the serine carboxypeptidase-like protein family in *Triticum aestivum* reveals *TaSCPL184-6D* is involved in abiotic stress response

**DOI:** 10.1186/s12864-021-07647-6

**Published:** 2021-05-15

**Authors:** Xiaomin Xu, Lili Zhang, Wan Zhao, Liang Fu, Yuxuan Han, Keke Wang, Luyu Yan, Ye Li, Xiao-Hong Zhang, Dong-Hong Min

**Affiliations:** 1 State Key Laboratory of Crop Stress Biology for Arid Areas and College of Agronomy, Northwest A&F University, Yangling, Shaanxi China; 2 Xinxiang Academy of Agricultural Sciences of He’nan Province, Xinxiang, China; 3 State Key Laboratory of Crop Stress Biology for Arid Areas and College of Life Sciences, Northwest A&F University, Yangling, Shaanxi China

**Keywords:** Serine carboxypeptidases-like protein, Genome-wide analysis, Drought stress, Salt stress, Wheat

## Abstract

**Background:**

The serine carboxypeptidase-like protein (SCPL) family plays a vital role in stress response, growth, development and pathogen defense. However, the identification and functional analysis of *SCPL* gene family members have not yet been performed in wheat.

**Results:**

In this study, we identified a total of 210 candidate genes encoding SCPL proteins in wheat. According to their structural characteristics, it is possible to divide these members into three subfamilies: CPI, CPII and CPIII. We uncovered a total of 209 *TaSCPL* genes unevenly distributed across 21 wheat chromosomes, of which 65.7% are present in triads. Gene duplication analysis showed that ~ 10.5% and ~ 64.8% of the *TaSCPL* genes are derived from tandem and segmental duplication events, respectively. Moreover, the Ka/Ks ratios between duplicated *TaSCPL* gene pairs were lower than 0.6, which suggests the action of strong purifying selection. Gene structure analysis showed that most of the *TaSCPL* genes contain multiple introns and that the motifs present in each subfamily are relatively conserved. Our analysis on *cis*-acting elements showed that the promoter sequences of *TaSCPL* genes are enriched in drought-, ABA- and MeJA-responsive elements. In addition, we studied the expression profiles of *TaSCPL* genes in different tissues at different developmental stages. We then evaluated the expression levels of four *TaSCPL* genes by qRT-PCR, and selected *TaSCPL184-6D* for further downstream analysis. The results showed an enhanced drought and salt tolerance among *TaSCPL184-6D* transgenic *Arabidopsis* plants, and that the overexpression of the gene increased proline and decreased malondialdehyde levels, which might help plants adapting to adverse environments. Our results provide comprehensive analyses of wheat *SCPL* genes that might work as a reference for future studies aimed at improving drought and salt tolerance in wheat.

**Conclusions:**

We conducte a comprehensive bioinformatic analysis of the *TaSCPL* gene family in wheat, which revealing the potential roles of *TaSCPL* genes in abiotic stress. Our analysis also provides useful resources for improving the resistance of wheat.

**Supplementary Information:**

The online version contains supplementary material available at 10.1186/s12864-021-07647-6.

## Background

Wheat (*Triticum aestivum*) is one of the most vital crops in the world, contributing a large amount of calories and protein to the global human diet [1, 2]. However, a variety of abiotic stresses seriously threaten the safety of wheat production. More than 50% of the world’s wheat producing areas are affected by drought stress [3], which is the main abiotic factor limiting the productivity of wheat in arid and semi-arid regions [4]. Moreover, drought and heat stress often occur simultaneously at sensitive growth stages reducing wheat yield by reducing the number or weight of grains [5]. With the global climate changes, the occurrence and severity of these events are also likely to increase [5]. In addition, out of 230 million hectares of irrigated land worldwide, 45 million hectares (19.5%) are threatened by salinization [6]. Soil salinization leads to reduced absorption of water and nutrients by plants [7], resulting in ion toxicity and oxidative damage to cells, thereby affecting their growth [8, 9]. In major wheat producing areas, the accumulation of lead is often accompanied by cadmium contamination [10]. Low concentration of cadmium in soil can inhibit normal cell division, reduce photosynthesis and damage the activity of antioxidant enzymes [11, 12], seriously threatening the yield and safety of crops. Therefore, mining stress related genes and identifying their functions are of great significance for the cultivation of stress-resistant wheat varieties. Studies have shown that *SCPL* genes play an important role in crop stress resistance. Therefore, it is of great significance to study the *SCPL* genes in wheat.

The *SCPL* genes belong to the S10 subfamily of the SC family [13, 14], which includes a highly conserved α/β hydrolase tertiary structure [15–18]. SCPL proteins contain a conserved triplet consisting of three amino acid residues: a serine, an aspartate and a histidine (Ser-Asp-His) [17, 18]. These three amino acid residues are located in different positions within the primary structure but in relative proximity to one another, relying on the folding of the polypeptide chains in order to form the conserved triplet in the tertiary structure [19]. This enables the SCPL proteins to bind to the substrate and cleave the carboxy terminal peptide bond of its protein or peptide substrate [20]. In addition, SCPL proteins have an oxygen ion hole that participates in the stabilization of the substrate-enzyme intermediate during the hydrolysis process [17]. Most SCPL proteins share common structural features, including four evolutionarily conserved domains that are involved in substrate binding and catalysis, a signal peptide sequence for intracellular transport or secretion, and multiple N-linked glycosylation sites [21, 22]. SCPL proteins are active under acidic pH conditions [13] and react during the proteolysis process [23–26].

The *SCPL* gene family has associated with biotic and abiotic stress responses. A type I SCP gene was identified in tomato (*Lycopersicon esculentum* Mill.) as one of the “late wound-inducible genes” based on its induced expression by wounding, systemin and methyl jasmonate (MeJA) [27]. The gene *OsBISCPL1* was significantly overexpressed in rice leaves that were treated with defense-related signaling molecules, such as salicylic acid (SA) and jasmonic acid (JA), or infected with magnaporthe grisea [28]. In addition, *Arabidopsis* plants overexpressing *OsBISCPL1* also showed an increased tolerance to oxidative stress, indicating that the gene may be involved in the regulation of defense responses against oxidative stress and pathogen infection [28]. In *Arabidopsis thaliana*, *SNG1* and *SNG2* act as acyltransferases and participate in the biosynthesis of sinapic acid esters, which has ultraviolet protection and antioxidant effects [29–32]. In addition, when respond to a variety of abiotic stresses, including drought, salinity, light, nitrogen and phosphorus deficiency, and suboptimal or supra-optimal temperatures, anthocyanins are also commonly induced in plants [33–39]. The roles of anthocyanins in abiotic stress include stress signaling [40, 41], photoprotection [42, 43], ROS quenching [44, 45]. In *Arabidopsis*, the gene AT2G23000 encode a sinapoyl-Glc:anthocyanin acyltransferase that is required for the synthesis of sinapoylated anthocyanins [46]. And both the serine carboxypeptidase-like 18 and the serine carboxypeptidase-like 18 isoform X3 are presumed to be involved in the biosynthesis of sinapoyl anthocyanin in *Dendrobium officinale* [47]. Finally, *SCPL* genes are also known to participate in the mobilization of storage proteins during seed germination [26, 48], the transformation of brassinolide signals [28, 49], the metabolism of herbicides [50], and to influence malting quality [51].

Whole-genome analysis of the *SCPL* gene family has been previously performed on a variety of plants. These studies have allowed the identification of 71 putative *SCPL* genes in rice (*O. sativa*), 54 in *Arabidopsis* (*A. thalianna*), 57 in poplar and 47 in the tea plant (*Camellia sinensis*) [52–54]. Here, we conducted a comprehensive genome-wide analysis of *SCPL* gene family in wheat and identified a total of 210 *SCPL* genes. In order to shed light on *SCPL* genes evolution and function, we performed a phylogenetic analysis and identified their physical location in different chromosomes, orthologous relationships, gene structure and tissue-specific expression patterns. The insights provided in this study will contribute to a better understanding on the evolution of *SCPL* genes and their role in the regulation of growth, development and responses to abiotic stress in wheat plants.

## Results

### Identification of wheat *SCPL* genes

**Fig. 1:**
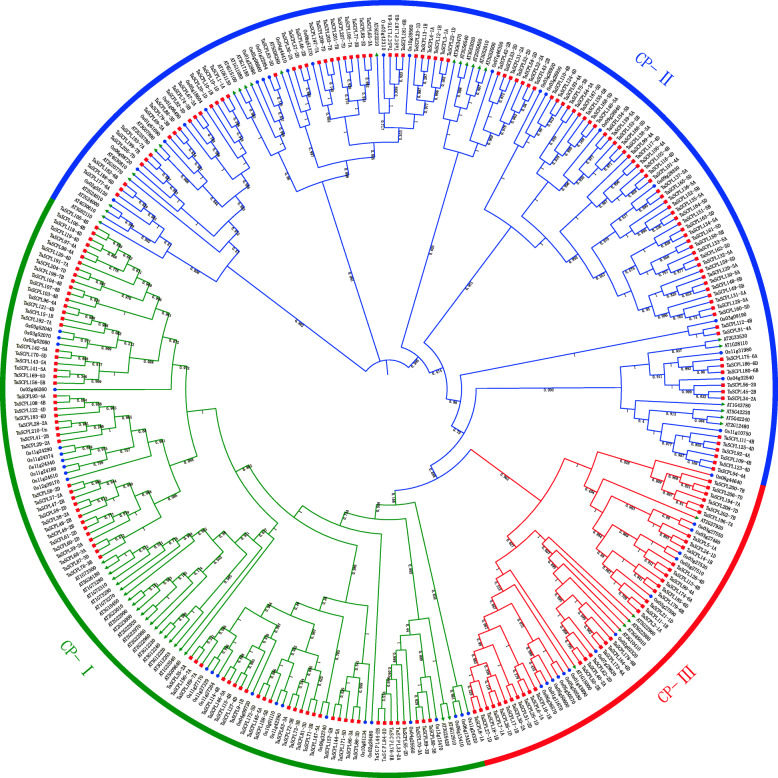
A phylogenetic tree of the SCPL proteins in wheat, rice and *Arabidopsis*. The complete amino acid sequences were aligned using ClustalX and a Maximum-likelihood method with Fasttree. The tree was divided into three subfamilies according to Shimodaira-Hasegawa test value and the amount of evolutionary distance estimated. These subfamilies are denoted by the different colors: CPI (green), CPII (blue) and CPIII (red). The three crops were marked with different colored shapes: wheat (red squares), rice (blue circles) and *Arabidopsis* (green triangles)

The process flow of this study is shown in Additional file 1: Figure S1. A total of 210 candidate *SCPL* genes were identified in wheat (Fig. 1). For convenience, these genes were termed *TaSCPL1-1A* through *TaSCPL210-Un* following their respective chromosomal locations. Even though these genes all have conserved SCPL protein domains, their size and physicochemical properties vary greatly. Detailed information on these candidate genes is summarized in Additional file 9: Table S1.

The transcripts (including the UTR and the CDS) of 210 *TaSCPL* genes ranged from 300 bp (*TaSCPL44-2B*) to 4553 bp (*TaSCPL124-4D*), with an average length of 1636 bp. The number of amino acids ranged from 99 (*TaSCPL44-2B*) to 563 amino acids (*TaSCPL62-2D*), and averaged 446. Furthermore, the molecular weight of the *TaSCPL* genes ranged from 11.42 kDa (*TaSCPL44-2B*) to 61.89 kDa (*TaSCPL62-2D*) with an average weight of 49.25 kDa. The isoelectric point (pI) values of these genes ranged from 4.64 (*TaSCPL159-5D*) to 9.44 (*TaSCPL182-6B*), with 80% members (168/210) exhibiting acidic pI values.

### Phylogenetic relationships and classification of TaSCPL proteins

**Fig. 2:**
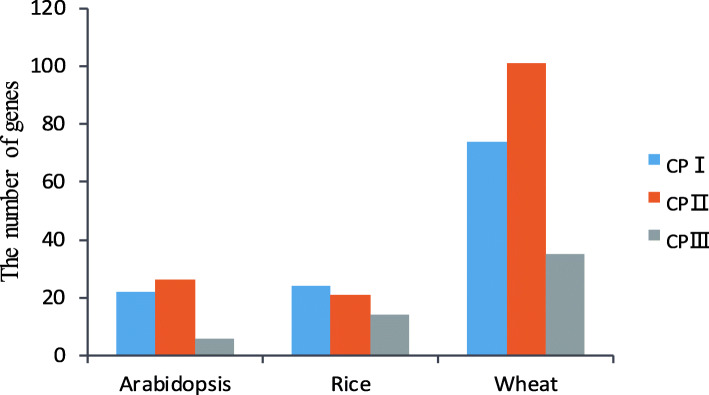
The number of *SCPL* genes found in each subfamily of *Arabidopsis*, rice and wheat

We constructed a phylogenetic tree on the SCPL proteins from wheat, rice and *Arabidopsis* in order to explore the evolutionary relationships among these proteins in the different species (Fig. 1). According to the structural features and the classification of the SCPL proteins in rice and *Arabidopsis* from previous studies [52], it was possible to divide the TaSCPL proteins into three distinct subfamilies, namely the Carboxypeptidase I (CPI), Carboxypeptidase II (CPII) and Carboxypeptidase III (CPIII). A higher number of proteins were distributed in the CPI and CPII subfamilies in the three species (Fig. 2). In the specific case of wheat, we found that 48.1% (101/210), 35.2% (74/210) and 16.7% (35/210) of the SCPL proteins were located in the CPII, CPI and CPIII subfamilies, respectively. As expected, the SCPL proteins within the same species tend to cluster on the same branch.

### Chromosomal location and identification of homoeologs

**Fig. 3:**
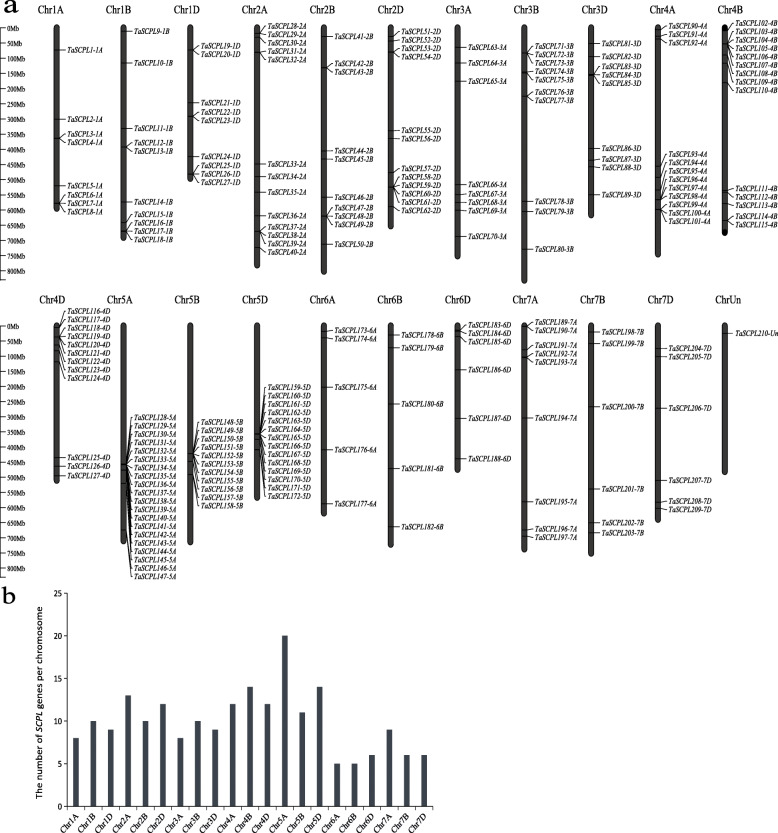
The distribution of 210 *TaSCPL* genes identified across different wheat chromosomes. **a** The physical location of 210 *TaSCPL* genes in wheat. The chromosome number (Chr1A–Chr7D) is indicated at the top of each chromosome. Gene names appear on the right close to their approximate location within the chromosomes. **b** The number of *SCPL* genes per chromosome

The precise locations of the *TaSCPL* genes on wheat chromosomes are listed in Additional file 9: Table S1. Most of these genes (209/210) were mapped to 21 chromosomes and revealed an uneven distribution in the genome, as shown in Fig. 3. There were a total of 27, 35, 27, 38, 45, 16 and 21 genes in chromosomes 1 to 7, respectively. The number of *TaSCPL* genes per chromosome ranged from 5 to 20, with clusters being observed on chromosomes 5A, 5B and 5D. Specifically, chromosome 5A contained the largest number of *TaSCPL* genes (20), followed by 4B and 5D (14), while both chromosomes 6A and 6B had the lowest (5). This suggests that the duplication of *TaSCPL* genes might have occurred during the formation of chromosomes 2, 4 and 5 in wheat. These results suggest that the evolution of the *TaSCPL* gene family occurred independently within the different sub-genomes.

In this study, we analyzed homoeologous groups in detail (Table 1 and Additional file 10: Table S2) and found that 35.8% of all wheat genes (i.e. in the current version of the wheat genome) were present in triads (homoeologous groups of 3) (IWGSC, 2018). In contrast, we observed that ~ 65.7% of the *TaSCPL* genes (138/210) were present in triads. Moreover, the proportion of homoeologous-specific duplications in *TaSCPL* genes was lower than that in all wheat genes (5.2% vs 5.7%). The loss of one homoeolog was less pronounced in the *TaSCPL* genes (8.6% vs 13.2%), as was the existence of orphans or singletons (9.5% vs 37.1%). Importantly, this high homoeolog retention rate can partly explain the existence of a higher number of *TaSCPL* genes in wheat than in both rice and *Arabidopsis*.

**Table 1:** Homoeologous *SCPL* genes in wheat

Homoeologous group (A:B:D)	All wheat genes	All wheat *SCPL* genes		
		Number of groups	Number of genes	% of genes
1:1:1	35.8%	46	138	65.7%
1:1:n/1:n:1/n:1:1, *n* > 1	5.7%	5	11	5.2%
1:1:0/1:0:1/0:1:1	13.2%	9	18	8.6%
Orphans/singletons	37.1%	–	20	9.5%
Other rations	8.0%	14	23	11.0%
	99.8%	–	210	100%

### Analyzing duplication events and natural selection

**Fig. 4:**
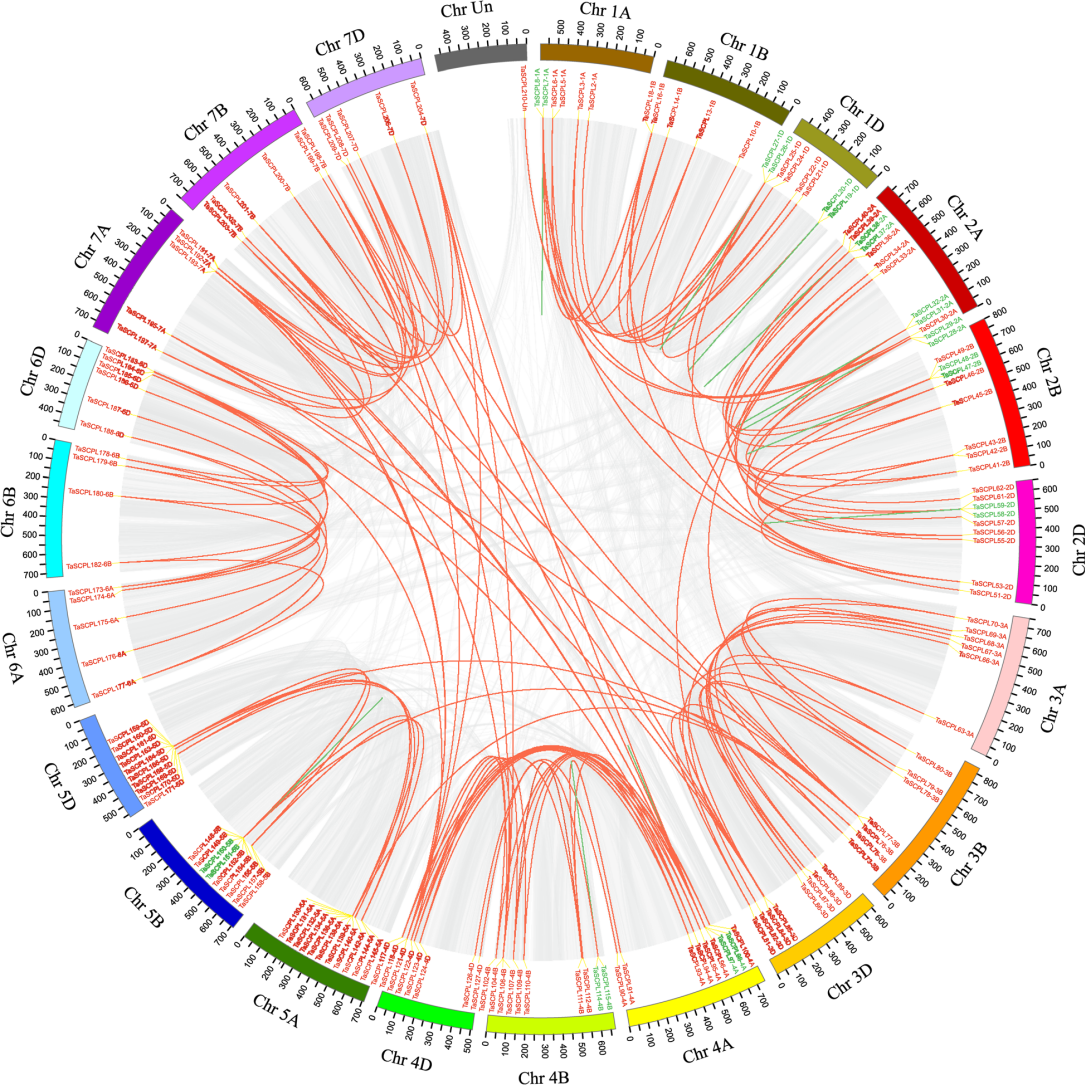
Collinearity analysis *SCPL* gene family in the wheat genome. WGD/segmental and tandem duplications of *TaSCPL* genes were mapped to their respective locations in the wheat genome and represented in a circular diagram using Circos. The panel shows each of the 21 wheat chromosomes displayed in a circle. Gray regions indicate the synteny blocks within the wheat genome. The red lines connect segmental duplicated *SCPL* gene pairs (shown in red). The green lines connect tandem duplicated *SCPL* gene pairs (shown in green). The different chromosomes are represented by different colors with the chromosome number displayed outside the track

To elucidate the evolutionary mechanisms behind the extension of *TaSCPL* genes, we evaluated tandem and segmental *TaSCPL* duplication events within the wheat genome. A total of 158 *TaSCPL* genes were located within syntenic blocks across different wheat chromosomes (Fig. 4 and Additional file 11: Table S3), forming 218 pairs of duplicated genes. We found that 54.4% (86/158) of the duplicated *TaSCPL* genes clustered on chromosomes 2, 4 and 5, which is consistent with the analysis described above. Statistical analysis showed that ~ 10.5% (22 out of 210) of the *TaSCPL* genes resulted from tandem duplication events (Additional file 11: Table S3), forming the following 11 pairs: *TaSCPL7-1A/8-1A*, *TaSCPL19-1D/20-1D*, *TaSCPL26-1D/27-1D*, *TaSCPL28-2A/29-2A*, *TaSCPL31-2A/32-2A*, *TaSCPL37-2A/38-2A*, *TaSCPL47-2B/48-2B*, *TaSCPL58-2D/59-2D*, *TaSCPL97-4A/98-4A*, *TaSCPL114-4B/115-4B* and *TaSCPL150-5B/151-5B*. In addition, 64.8% (136 out of 210) of the *TaSCPL* genes were associated with WGD/segmental duplication, which thus seems to represent one of the main contributing factors behind the significant expansion of *TaSCPL* genes in the wheat genome.

**Fig. 5:**
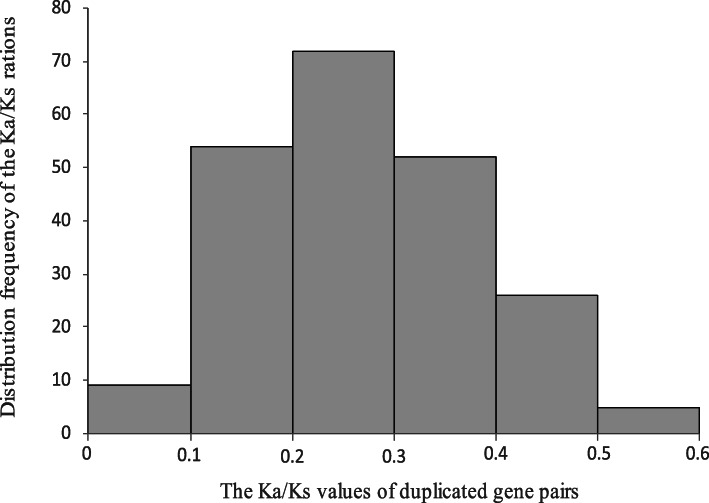
A histogram of the distribution of pairwise Ka/Ks ratios for duplicated *SCPL* gene pairs

To investigate the evolutionary forces acting on the 210 *TaSCPL* genes, we estimated Ka/Ks ratios for the different duplicated gene pairs (Additional file 11: Table S3). We found that the Ka/Ks ratios of all *TaSCPL* duplicated gene pairs were lower than 0.6, ranging from 0.067 (*TaSCPL193-7A*/*199-7B*) to 0.56 (*TaSCPL96-4A*/*121-4D*) and averaging 0.27. Moreover, the Ka/Ks ratios of 33% (72/218) of the duplicated gene pairs ranged from 0.2 to 0.3, 25% (54/218) ranged from 0.1 to 0.2, and 24% (52/218) ranged from 0.3 to 0.4 (Fig. 5). The Ka/Ks ratios of the 11 *TaSCPL* tandem duplicated gene pairs ranged between 0.21 and 0.44 (Additional file 11: Table S3). These observations suggest that duplicated *TaSCPL* genes have been evolving under purifying selection.

### Analyses on gene structure and conserved motifs

In order to gain a deeper understanding on the diversity of *TaSCPL* gene structure and function, we built a phylogenetic tree using the 209 TaSCPL protein sequences (except for *TaSCPL147-5A*, gene fragment loss may have occurred) (Additional file 2: Figure S2). We found that the structure of *TaSCPL* genes was relatively conserved within subfamilies, but differed between subfamilies. In the CPI subfamily, we found 4 genes with no introns, which ranged in number from 1 to 14 (with an average of 10). The number of introns of each gene in the CP II family ranged from 2 to 10 (with an average of 7), while only one gene did not contain intron. Finally, the number of introns per gene ranged from 1 to 12 (with an average of 7) in the CPIII subfamily, even though 10 out the 35 genes contained no intron.

We found that the motifs within TaSCPL proteins were generally well conserved, ranging in size from 11 to 80 amino acids in the 20 conserved motifs analyzed (Table 2). Specifically, the motifs of 1, 2, 3, 4, 5, 6, 8, 9 and 14 were present in almost all proteins (Additional file 2: Figure S2), while other motifs were specific to individual subfamilies in the phylogenetic tree. For example, motifs 10 and 12 were only detected in the CPI subfamily, motifs 11,13, 17 and 20 were specific to the CPII subfamily (motif 17 appeared in 3 CPI genes), and motifs 15 and 19 were solely found in the CPIII subfamily. These results indicated that TaSCPL proteins within the same subfamily often have similar motif composition. This is consistent with their relative phylogenetic relationships and suggests that the members of each subfamily are potentially associated with specific functions.

**Table 2:** List of the identified motifs in TaSCPL proteins

MOTIF	ID	WIDTH
1	PZYKGRPFYIAGESYAGHYVP	21
2	SWNKVANVJFLDSPAGVGFSY	21
3	LTFATVRGAGHTVPEYQPERA	21
4	YSGDHDSVVPVTGTRAWIRSLGLPV	25
5	KPLVLWLNGGPGCSS	15
6	GVPFDQYTGYVTVDEENGRALFYYFVEAE	29
7	WGHGIISDZLYEAITKNCDFD	21
8	VGDNRTALDAYVFLVKWFERF	21
9	GPLINLKGYLVGNPLTD	17
10	CRTYGYYLSYFWANBNMTRDALGIKKGTVGEWVR	34
11	YLNRPDVQKALHANTTGW	18
12	CNKDLPYTHDIPSSIKYHRNLTTRGYRAL	29
13	NWKDSPASMLPTJKWLIEAGJRVWV	25
14	MEELGPFRVNPDGKT	15
15	GVALGDSWISPEDFALSYAPLLYQVSRLDDNALDAANKLAATVKEQJAAGQFAAAEKSWTDLLDFIDQQSNSVDMYNFLL	80
16	WRPWHLDGQVA	11
17	AVADQSGAKEADRITALPGQP	21
18	AVADQSGAKEADRITALPGQP	15
19	LKIIPKBVTWEECSDAVYEALVNDFMKPRIPEVDELLRYGV	41
20	LVLFSSFLKGKLPPY	15

**Fig. 6:**
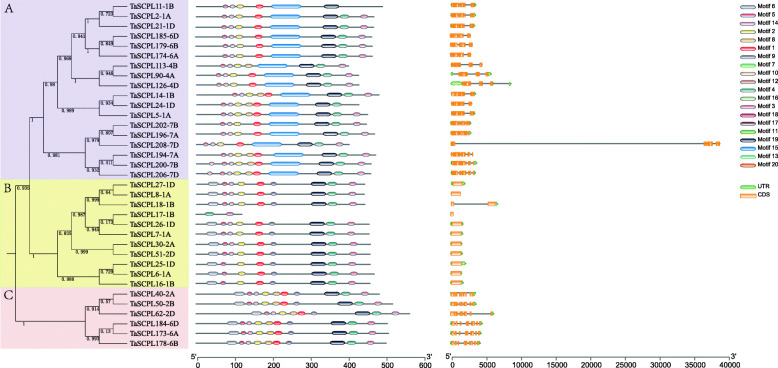
Phylogenetic relationship, gene structure and conserved motifs analysis of 35 CPIII *TaSCPL* genes. The phylogenetic tree was constructed using Fasttree. Twenty putative motifs are indicated as colored boxes. Introns and exons are indicated by black lines and orange boxes, respectively. The lengths of introns, exons and proteins of each gene can be inferred using the scale displayed at the bottom

Interestingly, our phylogenetic analysis revealed that almost all of the proteins within the same subfamily with similar gene and conserved motif structures clustered on the same branch. For example, the CPIII subfamily was divided into three branches termed A, B and C (Fig. 6). The 18 proteins of branch A had similar conserved motifs, with motif 15 being present in all genes. The majority of genes in the A branch contained a total of 11 introns, excepting for *TaSCPL113-4B* (12 introns), *TaSCPL14-1B*, *TaSCPL174-6A*, *TaSCPL179-6B* and *TaSCPL185-6D* (with 10 introns each). Except for one intron found in *TaSCPL18-1B*, the remaining 10 genes within branch B did not contain any introns. With the exception of *TaSCPL17-1B* (where a gene fragment loss may have occurred), the 10 members of the B branch possessed very similar conserved motifs. The 6 genes on branch C included 8 introns and their respective proteins contained the same conserved motifs. These results suggest that similar evolutionary events may affect the structure and function of these genes.

### Identification of *cis*-elements in the promoter region of *TaSCPL* genes

**Fig. 7:**
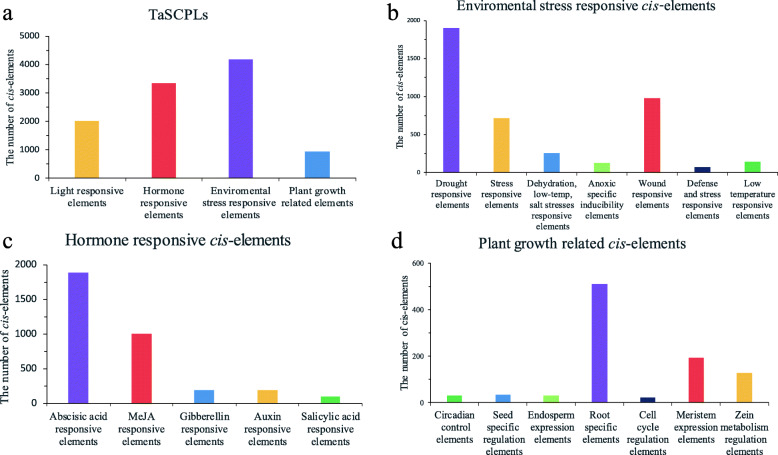
A 2000 bp region upstream of each *TaSCPL* gene was analyzed using PlantCARE. **a** The number of light responsive *cis*-elements, environmental stress responsive *cis*-elements, hormone responsive *cis*-elements, and plant growth related *cis*-elements in *TaSCPL* genes. **b** The number of environmental stress (drought, wound, low temperature, salt, dehydration, defense, and anaerobic) related *cis*-elements upstream of *TaSCPL* genes. **c** The number of different hormone (abscisic acid, MeJA, auxin, gibberellin and salicylic acid) responsive *cis*-elements in *TaSCPL* genes. **d** The number of plant growth (root, meristem expression and so on) related *cis*-elements in *TaSCPL* genes

We analyzed the promoter sequences of all *TaSCPL* genes using PlantCARE and found a huge number of *cis*-acting elements (Fig. 7 and Additional file 12: Table S4). The results showed that the majority of the uncovered *cis*-acting elements were environmental stress responsive elements (39.8%; 4188/10513), followed by hormone-responsive elements (31.9%; 3349/10513), light-responsive elements (19.3%; 2025/10513), and plant growth-related elements (9.0%; 951/10513) (Fig. 7a). Among the environmental stress responsive elements, most were associated with drought response (45.4%; 1900/4188), followed by wound (23.3%; 976/4188) and stress (17.1%; 716/4188) responses (Fig. 7b). Among the hormone-responsive elements, most constituted abscisic acid responsive elements (56.3%; 1886/3349), with a smaller proportion representing MeJA-responsive elements (30.0%; 1004/3349). These results demonstrated that *TaSCPL* genes are very likely associated with responses to abiotic stress, especially drought (Fig. 7c). In addition, among the identified elements that are related to plant growth, most were associated with root-specific responsive elements (53.6%; 510/951), suggesting that the *TaSCPL* gene family is also involved in root growth and development (Fig. 7d).

### Prediction of SSRs and miRNAs targeting *TaSCPL* genes

We identified 105 candidate gene based simple sequence repeat (cg-SSR) motifs from different regions of 210 wheat *SCPL* genes. The detailed information of the simple sequence repeat (SSR) was given in the Additional file 13: Table S5. Among all the identified SSRs, the largest number were trinucleotides (46.7%) followed by dinucleotides (40.0%). Among them, the most frequently repeated motif was (AGG/CCT)_5_, which accounted for 7.6% of the total motifs, followed by (AG/CT)_6_ (5.7%). A total of 24 different types of SSR motifs were identified, of which 8 types of SSR motifs appeared only once, and the remaining 16 types appeared 2–17 times. The most frequent occurrence was AG/CT (16.2%) followed by AC/GT (12.4%). The sub-genome level analysis revealed that 35.2% motifs were distributed in both the A and D sub-genome, while 27.6% motifs were distributed on the B sub-genome. Cg-SSRs were distributed on all the 21 wheat chromosomes, but the number of them was different (Additional file 3: Figure S3); the largest number of cg-SSRs was found on chromosome 2B (10.5%) and the smallest number (0.9%) was found on chromosomes 1B, 1D and 6B. Furthermore, some research indicated that SSR motifs within the genic regions might also be involved in regulating the expression of corresponding genes [55, 56]. Therefore, we designed 42 pairs of specific SSR primers (Additional file 14: Table S6), hoping to provide effective resources for trait mapping and crop breeding.

We also predicted putative microRNAs (miRNAs) targeting the *TaSCPL* genes by using the psRNATarget server [57]. The results showed that the *TaSCPL* genes were targeted by 4 different miRNAs (Additional file 15: Table S7) including tae-miR1130b-3p (MIMAT0035796), tae-miR1122a (MIMAT0005357), tae-MIR1127a (MIMAT0005362) and tae-miR1134 (MIMAT0005369). Among them, tae-miR1130b-3p belongs to the MiR1130 family, while the others belong to the MiR1122 family. These two miRNA families were conserved in crops and respond to a variety of biotic and abiotic stresses [58, 59]. Therefore, this study can provide help for understanding the mechanism of wheat stress resistance.

### Analysis of *TaSCPL* gene expression in wheat

**Fig. 8:**
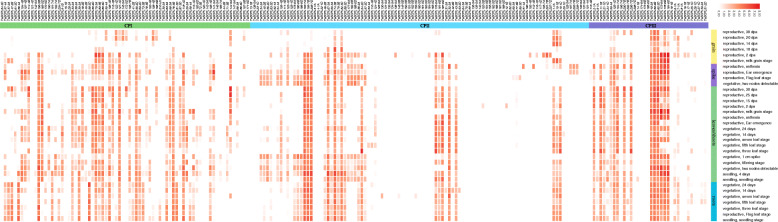
The expression of *TaSCPL* genes during wheat development (based on log_2_tpm). Expression values for all subfamilies were downloaded from the wheat expression database. The heatmap shows the expression levels of all *TaSCPL* genes in the different subfamilies (columns) and wheat developmental tissues/stages (rows). Genes and tissues are listed in Additional file 16: Table S8. Roots, leaves/shoots, spikes and grains are shown in blue, green, purple and yellow, respectively. The expression levels of each gene are indicated by the different color bars: red: higher expression; white: lower expression

In order to gain insight into the expression profiles of *TaSCPL* genes in different wheat tissues and periods, we downloaded expression data from the Wheat Expression Browser and generated a tissue-specific expression heatmap (Fig. 8 and Additional file 16: Table S8). Our analysis showed that 70.5% (148/210) of *TaSCPL* genes were expressed during one developmental stage, ranging from 1 to 8 Log_2_tpm (Log_2_tpm_max_) (Fig. 8 and Additional file 16: Table S8). The remaining 29.5% (62/210) of *TaSCPL* genes showed very low expression levels in all developmental stages (Log_2_tpm_max_ < 1) and were thus considered as unexpressed. Among the 74 genes of the CPI subfamily, 14.9% (11/74) were unexpressed, which could indicate that these genes underwent functional differentiation and redundancy. A variety of genes were highly expressed in the roots, leaves/shoots and spikes when comparing to grain. The CPII subfamily, which constitutes the largest clade, included a total of 42.6% (43/101) of unexpressed genes, indicating that genes in this subfamily might have experienced a stronger degree of functional differentiation and redundancy. Importantly, most other genes were expressed in all tissues. A few genes were specifically expressed in spikes, including *TaSCPL197-7A*, *TaSCPL203-7B* and *TaSCPL209-7D*, while others were expressed in the leaves/shoots and spikes, including *TaSCPL34-2A*, *TaSCPL45-2B* and *TaSCPL56-2D*. In CPIII family, 22.9% (8/35) of the genes showed very low to no transcripts. Some genes were expressed in various tissues, including six genes that displayed very high levels of transcription in the majority of tissues throughout wheat growth and developmental processes.

**Fig. 9:**
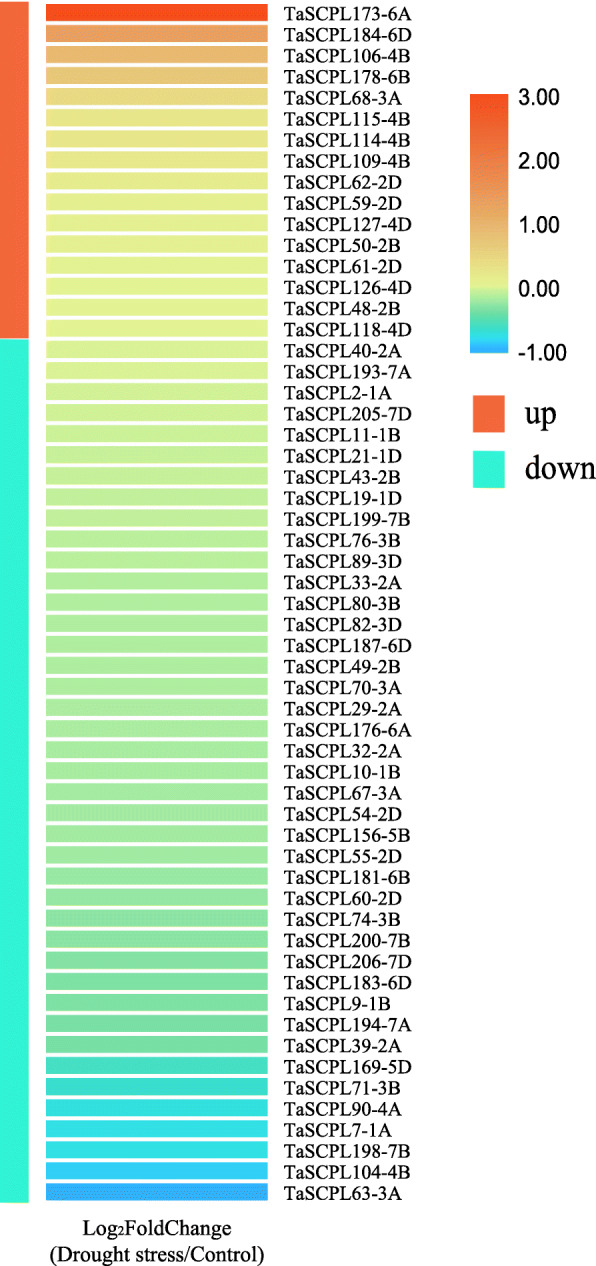
RNA-seq analysis of the wheat *SCPL* genes under drought stress. Orange and blue represent up- and down-regulation, respectively. The color intensity represents the fold change in the expression level compared to controls

In order to evaluate the expression of *TaSCPL* genes under abiotic stress, we downloaded the relative expression abundances of all *TaSCPL* genes in 7-day-old seedling leaves under drought stress from the Wheat Expression Browser (Additional file 17: Table S9). RNA-seq data suggested that a total of 57 *TaSCPL* genes were responsive to drought treatment (Fig. 9), of which 28.1% (16 out of 57) were up-regulated and 71.9% (41 out of 57) were down-regulated. These results indicated that abiotic stress can significantly induce multiple *TaSCPL* genes.

**Fig. 10:**
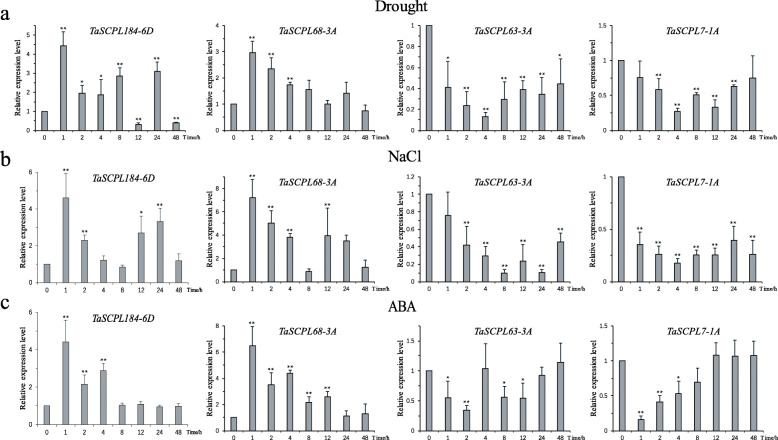
The qRT-PCR analyses of four wheat *TaSCPL* genes under drought, NaCl and ABA treatments. The β-actin gene was used as an internal control. The mean and SD were calculated from three biological replicates. The y-axis represents the relative expression level. The x-axis indicates the duration of treatment: 0, 1, 2, 4, 8, 12, 24, and 48 h

The expression patterns of four *TaSCPL* genes were then examined under drought, salt and ABA treatment using qRT-PCR (Fig. 10). The results obtained were consistent those of the RNA-seq experiment. The genes *TaSCPL184-6D* and *TaSCPL68-3A* showed a similarly up-regulated expression pattern under the three abiotic stress treatments. When compared to control samples, *TaSCPL184-6D* showed higher expression levels than *TaSCPL68-3A* under stress conditions. Specifically, *TaSCPL184-6D* showed the highest levels of expression after 1 h under drought and salt treatment and 24 h under the ABA treatment. *TaSCPL68-3A* reached its highest expression levels 1 h after the three treatments. Importantly, both *TaSCPL63-3A* and *TaSCPL7-1A* showed similarly down-regulated expression patterns under the three abiotic stress treatments. Finally, transcription analysis revealed that *TaSCPL184-6D* was significantly up-regulated under drought, salt and ABA treatments (Fig. 10), whereby it was chosen for downstream analyses.

### The overexpression of *TaSCPL184-6D* affects seed germination under drought and NaCl stress in *Arabidopsis*

In order to evaluate the functional role played by *TaSCPL184-6D* under drought or salt treatment, we used three homozygous T3 generation *TaSCPL184-6D*-overexpressing *Arabidopsis* lines (OE-1, OE-2 and OE-3). In order to examine seed germination, we used Murashige and Skoog (MS) mediums containing 8% polyethylene glycol 6000 (PEG6000), 10% PEG6000, 100 mM NaCl and 150 mM NaCl. We found no significant differences in the germination rate between wildtype (WT) and *TaSCPL184-6D*-overexpressing lines on a MS medium (Additional file 4: Figure S4, Additional file 5: Figure S5 and Additional file 19: Table S11). However, when PEG6000 was added to the MS medium, the germination of both WT and *TaSCPL184-6D*-overexpressing lines was inhibited, but the latter showed higher germination rates. The differences between WT and *TaSCPL184-6D*-overexpressing lines peaked 36 and 48 h after treatment with 8 and 10% PEG6000, respectively (Additional file 4: Figure S4). The germination rates of WT and *TaSCPL184-6D*-overexpressing lines was significantly also reduced on a MS medium containing NaCl, and WT seeds showed a slower germination speed and rate compared to *TaSCPL184-6D*-overexpressing lines (Additional file 5: Figure S5), especially under the 150 mM NaCl treatment condition.

### T*aSCPL184-6D* conferred increased drought and salt tolerance in *Arabidopsis*

To explore the function of *TaSCPL184-6D* under drought treatment, we conducted root length determination and seedling drought resistance studies. The 7-day-old WT and *TaSCPL184-6D*-overexpressing lines growing uniformly on a MS medium were transferred to another MS medium containing 10% PEG6000. We evaluated the phenotypic changes on these plants after 7 days of cultivation. No significant differences in root length and fresh weight were observed between WT and *TaSCPL184-6D*-overexpressing lines on the control MS medium, and plant growth followed normal trajectories (Additional file 6: Figure S6). However, under drought treatment with 10% PEG6000, all *TaSCPL184-6D*-overexpressing lines showed longer root lengths and heavier fresh weights than WT plants. Moreover, these plants exhibited a better growth trajectory than WT (Additional file 6: Figure S6 and Additional file 20: Table S12). To evaluate changes in drought resistance of three-week-old WT and *TaSCPL184-6D*-overexpressing lines planted in the soil, we counted their respective survival rates and measured the amounts of proline (Pro) and malondialdehyde (MDA) that were present (Additional file 7: Figure S7 and Additional file 20: Table S12). Our results showed that, after drought treatment, both the survival rates and the amount of Pro in the *TaSCPL184-6D*-overexpressing lines were significantly higher than that of WT. In contrast, the amount of MDA in *TaSCPL184-6D*-overexpressing lines was significantly lower than that of the WT.

We also conducted a further study on analyzing the effects salt treatment on *TaSCPL184-6D*-overexpressing lines. We found that all transgenic lines showed significantly longer root length and heavier fresh weight than the WT after 150 mM NaCl treatment (Additional file 6: Figure S6 and Additional file 20: Table S12). We also showed that the survival rates and the amount of Pro of the *TaSCPL184-6D*-overexpressing lines were significantly higher than those of WT when evaluating salt tolerance at the seedling stage. In contrast, we observed that the amount of MDA was significantly lower than that of the WT (Additional file 8: Figure S8 and Additional file 20: Table S12).

## Discussion

The *SCPL* gene family plays an important functional role in plants. Whole-genome analysis of *SCPL* genes has been performed in a variety of plants and allowed for the identification of 71 putative *SCPL* genes in rice (*O. sativa*), 54 in *Arabidopsis* (*A. thalianna*), 57 in poplar and 47 in the tea plant (*Camellia sinensis*) [52–54]. However, no systemic analysis of the *SCPL* gene family has been reported in wheat. Therefore, this study aimed to conduct a comprehensive and systematic analysis of the wheat *SCPL* gene family, to explore the phylogeny and evolution of *SCPL* genes in wheat, and to investigate the phenotypes of *Arabidopsis* plants overexpressing the *TaSCPL184-6D* gene under drought and salt stress.

A total of 210 *SCPL* genes were identified in wheat and divided into three subfamilies (Fig. 1). The number of genes in the wheat *SCPL* family is ~ 3.9x, 3x, 3.7x and 4.5x higher than in *Arabidopsis*, rice, poplar and tea, respectively. Approximately 65.7% (138 out of 210) of the *TaSCPL* genes were present in triads, whereas this proportion is ~ 35.8% across all wheat genes (IWGSC, 2018). Therefore, the high homologue retention rate could partly explain why the number of *SCPL* genes is higher in wheat than in other species. The phylogenetic analysis showed that wheat, tea, poplar and *Arabidopsis* have more genes in the CPI and CPII subfamilies. This indicates that the *SCPL*s in these different species might be undergoing conservative evolution [52–54].

The physical location of the *SCPL* genes in different species is unevenly distributed across the chromosomes. Specifically, 23.4, 19.3, 29.6 and 16.9% of the *SCPL* genes were located on Scaffold2990 in tea plants, 1.MAP in poplar, Chr.4 in *Arabidopsis thaliana* and Chr.11 in rice, respectively. However, some chromosomes contained only a single *SCPL* gene in these species [52–54]. We showed that the distribution of *SCPL* genes in wheat is distributed across 21 chromosomes. There was a total of 20 *SCPL* genes on chromosome 5A, but only 5 on chromosomes 6A and 6B. In addition, the 9 chromosomes of groups 2, 4 and 5 not only contained more genes (56.2%, 118/210) but also contained 54.4% (86/158) of duplicated genes, which are often observed in plant genomes and are produced by tandem and segmental duplications or polyploidization [60]. The expansion of the *SCPL* gene family in wheat mainly occurred through segmental duplications, which was consistent with the observations for the CsSCPL-IA subfamily in tea plants [54]. The Ka/Ks values of the 218 duplicated gene pairs in the wheat *SCPL* gene family were all lower than 0.6, indicating that these genes are evolving under strong purifying selection.

Transcription factors (TFs) can regulate plant functions, including responses to hormones and environmental factors, cell differentiation and organ development, by regulating gene expression [61]. The analysis of *cis*-elements located in the promoter region of *SCPL* genes showed that the most hormone-responsive elements in wheat were the ABA- and the MeJA-responsive *cis*-elements. In contrast, the most relevant poplar *cis*-elements were the MeJA-responsive elements [53]. Among the environmental stress responsive elements, the most important were those related to drought in both wheat and poplar [53]. These results indicate that the *SCPL* gene family is likely involved in responses to drought stress through the ABA or the MeJA pathways.

In recent years, due to global climate change, a variety of abiotic stresses have become more frequent, in particular drought and salt stress. Abiotic stress affects plant growth and development by changing their physiology and metabolism, which ultimately leads to the decline of crop yield and quality [62, 63]. In this study, we cloned the candidate *TaSCPL184-6D* wheat gene and overexpressed it in *Arabidopsis thaliana* in order to investigate its role in mediating plant responses to abiotic stress. We found that, when compared to the WT, transgenic *Arabidopsis* plants cultivated under drought or salt stress showed a higher germination and survival rates, as well as an increased root length. Moreover, Pro is involved in maintaining the dynamic plant balance in the face of adverse conditions [64]. Salt stress can cause oxidative and hyperosmotic damage to the cell membranes [65], and the amount of MDA can reflect the degree of damage suffered by plants. Under drought or salt treatments, we found a higher accumulation of Pro and a lower accumulation of MDA in *TaSCPL184-6D* transgenic *Arabidopsis* plants when compared to WT. This suggests that Pro might directly contribute to an enhanced tolerance of *TaSCPL184-6D* transgenic plants to drought and salt stress (Additional file 7: Figure S7 and Additional file 8: Figure S8). These physiological changes suggest that *TaSCPL184-6D* plays an active role in plant response to adverse environmental conditions.

## Conclusion

We identified a total of 210 *SCPL* genes from the wheat genome, which were termed from *TaSCPL1-1A* to *TaSCPL210-Un* in accordance to their chromosomal positions, and performed comprehensive and systematic analysis on these genes. Our results showed that the *TaSCPL184-6D* gene, a member of the CPIII subfamily, improves the tolerance of *Arabidopsis* plants to drought and salt stresses. This study provides valuable insights for further understanding the evolutionary mechanisms behind wheat *SCPL* genes, and constitutes an important reference for the genetic improvement of wheat resistance.

## Methods

### Identification of wheat *SCPL* genes

We downloaded the protein sequences from the Ensembl Plants database (http://plants.ensembl.org/index.html) [66] and obtained the Hidden Markov Model (HMM) profile of the SCPL conservative domain (PF00450) from Pfam (https://pfam.xfam.org) [67–69]. The wheat *SCPL* gene family was subsequently identified in accordance to previous methods [70, 71]. The identified candidate *SCPL* genes were submitted to the NCBI protein Batch CD-search database (http://www.ncbi.nlm.nih.gov/Structure/bwrpsb/bwrpsb.cgi) [72] and the SMART database (https://smart.embl-heidelberg.de/) [73] in order to eliminate the genes lacking the conserved domain. After this verification, all of the candidate *SCPL* genes were identified in the wheat genome. The ExPASY website (https://www.expasy.org/) was used to predict the physicochemical parameters of the SCPL proteins, including molecular weight (Mw) and theoretical pI [74].

### Phylogenetic analysis and classification of TaSCPL proteins

A multiple sequence alignment of the SCPL sequences was generated using ClustalX [75] and removed Gap-only columns. The approximately-maximum-likelihood phylogenetic trees was constructed by the FastTree version 2.1.3 [76] using the JTT (Jones-Taylor-Thornton) model [77]. The “CAT” approximation [78] with 20 rate categories was used to account for the varying rates of evolution across sites. And the Shimodaira-Hasegawa test (1000 resamples) [79] was used to compute local support values. The trees were visualized using Figtree version 1.4.3.

### Chromosomal location and identification of homoeologs

The *SCPL* genes were mapped against the 21 wheat chromosomes using positional information acquired from the Ensembl Plants database and the Mapchart software [80]. Homoeologous genes were identified using the phylogeny [81] and the Ensembl Plants database.

### Gene duplication and evolutionary selection analysis

Segmental and tandem duplication events were determined according to previously described methods [71, 82]. Briefly, all TaSCPL proteins were searched using BLASTp (E-value < 10^− 10^) and a pair of duplicated TaSCPL proteins were defined based on the following three criteria: 1) the alignment covered > 80% of the longer gene; 2) the aligned region had an identity > 80%; 3) only one duplication event was counted for the genes which were tightly linked [83, 84]. Tandem repeat events were divided according to the chromosomal position of each duplicated gene. The Circos software was used to visualize the duplicated gene pairs in the *T.astivum* genome (Fig. 4) [85].

To assess the mode of selection acting on each *TaSCPL* duplicated gene pair, we calculated the rates of synonymous (Ks) and non-synonymous (Ka) substitutions, and their respective ratio, between two duplicated *TaSCPL* genes using the TBtools software [86]. Detailed information on the duplicated gene pairs can be found in Additional file 11: Table S3. A Ka/Ks ratio > 1 is often associated with positive selection, a Ka/Ks = 1 represents neutrality, and a Ka/Ks ratio of < 1 may represent purifying selection [87].

### Gene structure and conserved motifs analysis

The exon/intron structure information for each gene was obtained from the Ensembl Plants database (http://plants.ensembl.org/index.html). The exon-intron substructure map was produced using TBtools [86]. The conserved motifs of each SCPL protein were examined using the Multiple Expectation Maximization for Motif Elicitation program (MEME, http://meme-suite.org/tools/meme) [88] and visualized by TBtools [86]. The number of motifs was set at 20 with motif width ranges from 6 to 100 (inclusive) amino acids. Any number of repetitions were considered during the verification, and the other parameters were default.

### Identification of *cis*-elements in the promoter region of *TaSCPL* genes

In order to infer possible biological functions and the transcriptional regulation of the *TaSCPL* genes, the 2000 bp region upstream of the translation initiation site was used as the promoter region in order to identify *cis*-elements by submitting them to the PlantCARE database (http://bioinformatics.psb.ugent.be/webtools/plantcare/html/) [89].

### Prediction of cg-SSRs and miRNAs targeting *TaSCPL* genes

Cg-SSRs were developed inside the genomic sequences of *TaSCPL* genes using MISA [90]. The screening parameters were: dinucleotides, trinucleotides, tetranucleotides, pentanucleotides and hexanucleotides were ≥ 6, 5, 5, 4 and 4, respectively. The specific primers for SSRs were designed by Primer3 [91] and screened by e-PCR [92]. The potential MiRNAs targeting *TaSCPL* genes were predicted according to previously described methods [93]. Briefly, the psRNATarget server [57] was used to predict MiRNA, and MirBase 22.0 (http://www.mirbase.org/) [94] was used to query the specific information of miRNAs.

### Expression analysis of *TaSCPL* genes

To study the expression of *TaSCPL* genes in different tissues and the response to drought, we obtained transcriptome data from the Wheat Expression browser (http://www.wheat-expression.com) [95]. Log_2_(transcripts per million) (log_2_tpm) values were used to estimate gene expression in different tissues (root, leaf/shoots, spikes and grains) and under drought treatment. The fold changes in expression levels relative to the control were used to generate a heatmap for NaCl treatment. The log_2_tpm values of *TaSCPL* genes in different tissues and under drought stress treatment are shown in Additional file 16: Tables S8 and Additional file 17: Tables S9. The TBtools software was used to visualize the expression levels of the *TaSCPL* genes in the heatmap [86].

### Plant materials, growth conditions and stress treatments

Seeds from the wheat variety Chinese Spring were used for gene expression analysis. The treatment methods of plant materials refer to the previous literature and make some modifications [96, 97]. The seeds grew in a greenhouse under a temperature of 22 / 20 °C of day and night, and with a photoperiod of 16 h light / 8 h darkness. The 7-day-old seedlings underwent stress and hormone treatments. The seedlings were put on paper filters to perform drought stress, and were irrigated with a 100 mM NaCl solution to simulate salt treatment. For hormone treatments, the seedlings were exposed to solutions with 100 μM ABA, 100 μM MeJA, 100 μM BR and 100 μM GA. Seedling leaves from all treatments and controls were carefully harvested at 0, 1, 2, 4, 8, 12, 24 and 48 h, immediately frozen in liquid nitrogen and stored at − 80 °C for subsequent analysis.

The *Arabidopsis* Col-0 plants were used for phenotypic assays. The growth conditions of the *Arabidopsis* plants were the same as those aforementioned for wheat. In order to obtain *TaSCPL184-6D* transgenic *Arabidopsis*, we linked the open reading frame (without the termination codon) of the *TaSCPL184-6D* gene to the pCAMBIA1302 vector. The recombinant plasmid was verified by sequencing, transformed into the *Agrobacterium tumefaciens* strain GV3101, and then transformed into *Arabidopsis* Columbia-0 (Col-0) according to a previously described flower immersion method [98]. The positive *TaSCPL184-6D* transgenic plants were identified by PCR and cultured to the T_3_ generation. The expression levels of *TaSCPL184-6D* in the T_3_ generation transgenic lines were determined by qRT-PCR, and the three lines with the highest expression levels were selected for further identification of stress resistance.

The WT and *TaSCPL184-6D* transgenic *Arabidopsis* were used to assess drought and salt tolerance. For germination analysis, the seeds of WT and *TaSCPL184-6D*-overexpressing lines were surface sterilized and sown on a MS medium containing PEG6000 (8 and 10%) (m/v) and NaCl (100 mM and 150 mM). Subsequently, 3 days after vernalization, the seeds were transferred to normal conditions for germination. When the radicle broke through the seed coat, the seed was considered as germinated. The germination rates were counted every 12 h. For root length determination, 7-day-old uniformly germinated seeds were transferred to a MS media with 10% PEG6000 or 150 mM NaCl. The root length was evaluated after treatment for 7 days. To assess drought tolerance in the soil, 3-week-old seedlings were kept dry for 2 weeks and then rewatered for 3 days. For salt treatment, the seedlings grown under normal conditions were irrigated with 100 mM NaCl solution for 7 days and then watered for another 3 days. We recorded the survival rates and performed three independent biological replicates.

### RNA extraction and quantitative real-time PCR

Total RNA was isolated from wheat leaves treated under various stress conditions using the Trizol reagent (TaKaRa, Japan). The FastKing RT Kit (With gDNase) (TIANGEN, China) was used to remove the contamination of genomic DNA and perform cDNA synthesis following the manufacturer’s instructions. Quantitative real-time PCR (qRT-PCR) was performed using the SuperReal PreMix Color (SYBR Green) (TIANGEN, China) and the QuantStudio 7 Flex Real-Time PCR system (ThermoFisher, USA). The wheat β-actin gene (GenBank accession number AB181991.1) was used as an internal reference for all qRT-PCR analysis. We performed three independent replicates for each treatment. The relative expression levels of each gene was calculated based on the 2^-△△CT^ value [99]. The specific primers used for qRT-PCR are listed in Additional file 18: Table S10.

### Measuring the amount of MDA and pro

To measure the amount of Pro and MDA, 3-week-old seedlings of WT and transgenic *Arabidopsis* plants were subjected to drought for 2 weeks or salt stress for 1 week. The corresponding assay kit (Cominbio, Suzhou, China) was then used to measure the amount of Pro and MDA in the leaves. A total of three repetitions were implemented in each measurement.

### Statistical analysis

The aforementioned experiments were repeated 3 times, independently, in order to obtain sufficient data to perform statistical analyses. The values are shown as mean ± standard deviation (SD). An ANOVA test was used for statistical analysis. The significance levels were defined as ∗ (*P* < 0.05); and ∗∗ (*P* < 0.01).

## Supplementary Information

**Additional file 1: Figure S1.** The research process of this study.

**Additional file 2: Figure S2.** Phylogenetic relationship, gene structure and conserved motifs analysis of 209 *TaSCPL* genes.

**Additional file 3: Figure S3.** The number of SSRs per chromosome.

**Additional file 4: Figure S4.** The overexpression of *TaSCPL184-6D* increased the germination rate of seeds under PEG6000 treatment. **a** The phenotypes of WT and *TaSCPL184-6D* transgenic *Arabidopsis* seeds under 8 and 10% PEG6000 treatments. **b** The germination rates of WT and *TaSCPL18*4*-6D* transgenic *Arabidopsis* seeds at different time points on MS medium. **c** The germination rates under 8% PEG6000 treatment. **d** The germination rates under 10% PEG6000 treatment. A total of three biological replicates were performed. The error bars indicate the standard deviation (SD) of the three replicates.

**Additional file 5: Figure S5.** The overexpression of *TaSCPL184-6D* increased the germination rate of seeds under NaCl treatment. **a** The phenotypes of WT and *TaSCPL184-6D* transgenic *Arabidopsis* seeds under 100 mM and 150 mM NaCl treatments. **b** The germination rates of WT and *TaSCPL18*4*-6D* transgenic *Arabidopsis* seeds at different time points on MS medium. **c** The germination rates under 150 mM NaCl treatment. **d** The germination rates under 10% PEG6000 treatment. A total of three biological replicates were performed. The error bars indicate the SD of the three replicates.

**Additional file 6: Figure S6.** The overexpression of *TaSCPL184-6D* enhanced the tolerance to drought and salt stresses in Arabidopsis. **a** Root length assays of wild-type and *TaSCPL184-6D*-overexpressing plants on a MS medium. **b** Root length assays under 10% PEG6000 treatment. **c** Root length assays under 150 mM NaCl treatment. **d** Tool root length under 10% PEG6000 treatment. **e** Tool root length under 150 mM NaCl treatment. **f** Fresh weight under 10% PEG6000 treatment. **g** Fresh weight under 150 mM NaCl treatment.

**Additional file 7: Figure S7.** The overexpression of *TaSCPL184-6D* enhanced drought tolerance in *Arabidopsis*. **a** The drought tolerance phenotypes of WT and *TaSCPL184-6D* transgenic *Arabidopsis* in soil. Three-week-old seedlings of WT and *TaSCPL184-6D*-overexpressing lines were dehydrated for 2 weeks and then rewatered for 3 days. **b** Statistical analysis of survival rates. **c** The amount of Pro. **d** The amount of MDA. Data is shown as the mean ± SD of three independent replicates. Significant differences were observed using a Student’ s t test (*p < 0.05, **p < 0.01).

**Additional file 8: Figure S8.** The overexpression of *TaSCPL184-6D* enhanced the tolerance to NaCl tolerance in *Arabidopsis*. **a** NaCl tolerance phenotypes of WT and *TaSCPL184-6D* transgenic *Arabidopsis* in soil. Three-week-old seedlings of WT and *TaSCPL184-6D*-overexpressing lines were stressed for 7 days and then rewatered for 3 days. **b** Statistical analysis of survival rates. **c** The amount of Pro. **d** The amount of MDA.

**Additional file 9: Table S1**. The characteristics of 210 wheat *SCPL* genes.

**Additional file 10: Table S2.** Homoeologous *SCPL* genes in wheat.

**Additional file 11: Table S3.** The Ka/Ks ratios of duplicated *SCPL* gene pairs.

**Additional file 12: Table S4.** C*is*-acting elements in the promoter region of *TaSCPL* genes.

**Additional file 13: Table S5.** The characteristics of 105 SSRs.

**Additional file 14: Table S6.** The information of SSR primers.

**Additional file 15: Table S7.** The detailed information of predicted miRNAs.

**Additional file 16: Table S8.** The log_2_tpm values of all *TaSCPL* genes in the different subfamilies and wheat developmental tissues/stages.

**Additional file 17: Table S9.** The log_2_tpm values of all *TaSCPL* genes under drought stress treatment.

**Additional file 18: Table S10.** The primers used in this study.

**Additional file 19: Table S11.** The germination rates of seeds in transgenic *Arabidopsis* plants under PEG6000 and NaCl treatments.

**Additional file 20: Table S12.** Total root length, fresh weight, and the amount of PRO and MDA.

## Data Availability

All data generated or analyzed during this study are included within the article and its additional files. The phylogenetic data in our manuscript has been deposited into Treebase database with the access URL is http://purl.org/phylo/treebase/phylows/study/TB2:S27753.

## Abbreviations

SCPL: Serine carboxypeptidase-like protein
SA: Salicylic acid
JA: Jasmonic acid
MeJA: Methyl jasmonate
GA: Gibberellin
MS: Murashige and Skoog
Ka: Non-synonymous
Ks: Synonymous
SSR: Simple sequence repeat
Pro: Proline
MDA: Malondialdehyde
PEG6000: Polyethylene glycol 6000
Mw: Molecular weight
pI: Isoelectric point
SD: Standard deviation
