# Supplementary figures and images for: Genome-wide analysis of the serine carboxypeptidase-like protein family in Triticum aestivum reveals TaSCPL184-6D is involved in abiotic stress response
Source: BMC Genomics. 2021 May 15;22:350. doi: 10.1186/s12864-021-07647-6 (PMC8126144; doi:10.1186/s12864-021-07647-6)

# Additional file 1:Figure S1. The research process of this study.

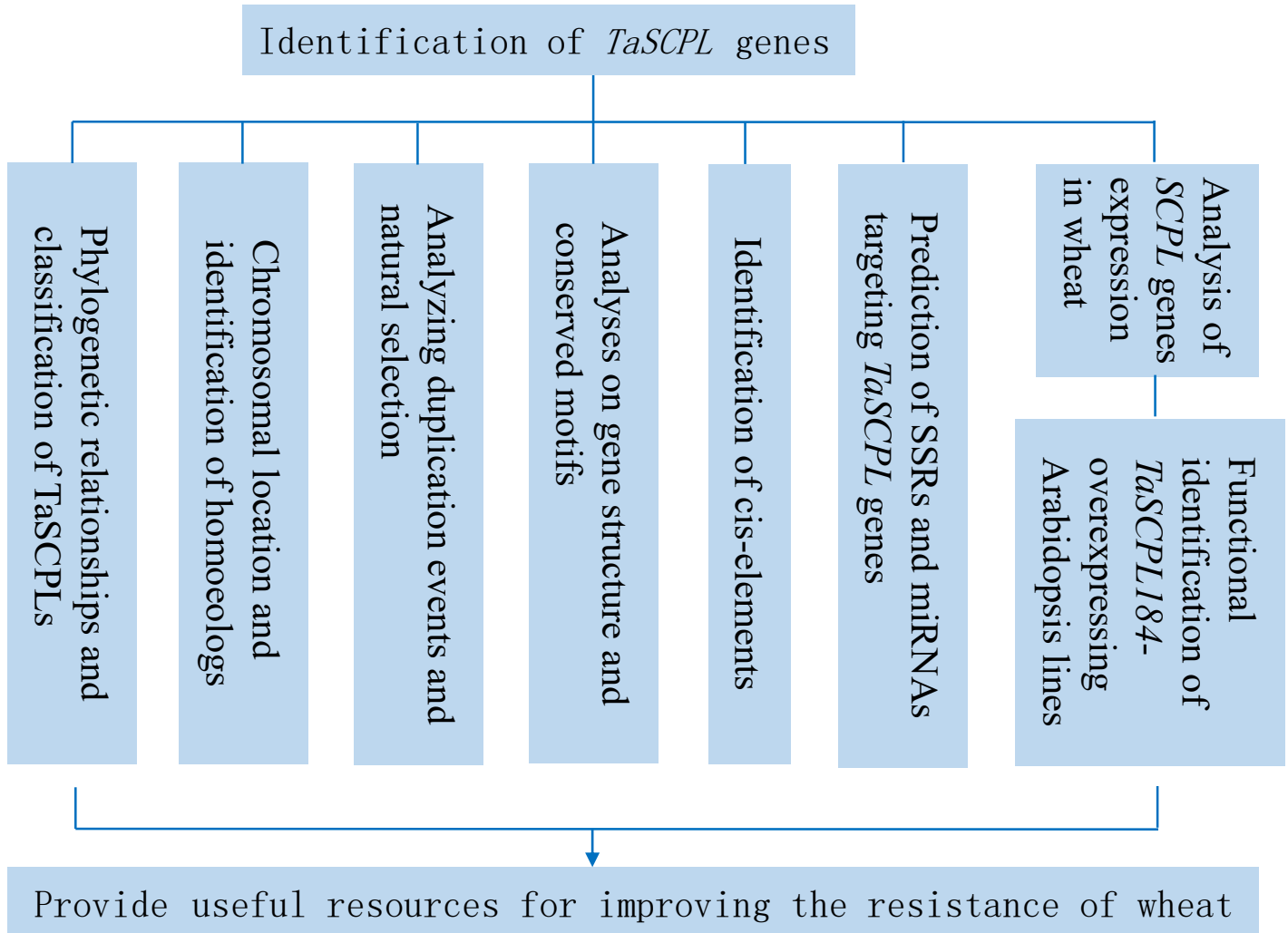

Supplement: Supplementary file 1 — Additional file 1: Figure S1. The research process of this study. [file 12864_2021_7647_MOESM1_ESM.pdf]

**Additional file 3: Figure S3.** The number of SSRs per chromosome.

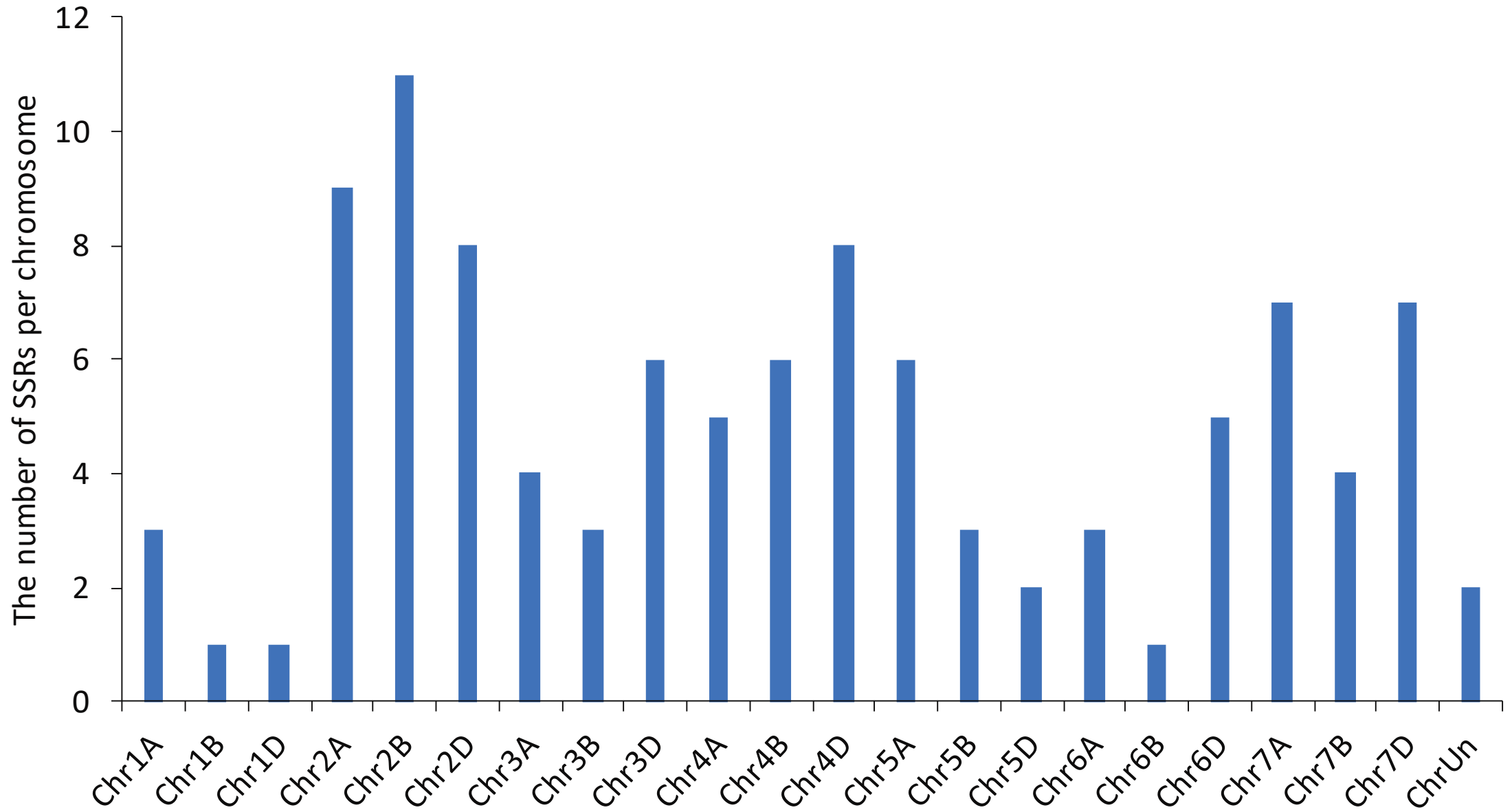

Supplement: Supplementary file 3 — Additional file 3: Figure S3. The number of SSRs per chromosome. [file 12864_2021_7647_MOESM3_ESM.pdf]
