# Supplementary material for: Genome-wide analysis of the serine carboxypeptidase-like protein family in Triticum aestivum reveals TaSCPL184-6D is involved in abiotic stress response
Source: BMC Genomics. 2021 May 15;22:350. doi: 10.1186/s12864-021-07647-6 (PMC8126144; doi:10.1186/s12864-021-07647-6)

Additional file 2:Figure S2. Phylogenetic relationship, gene structure and conserved motif analysis of 209 *TaSCPL* genes.

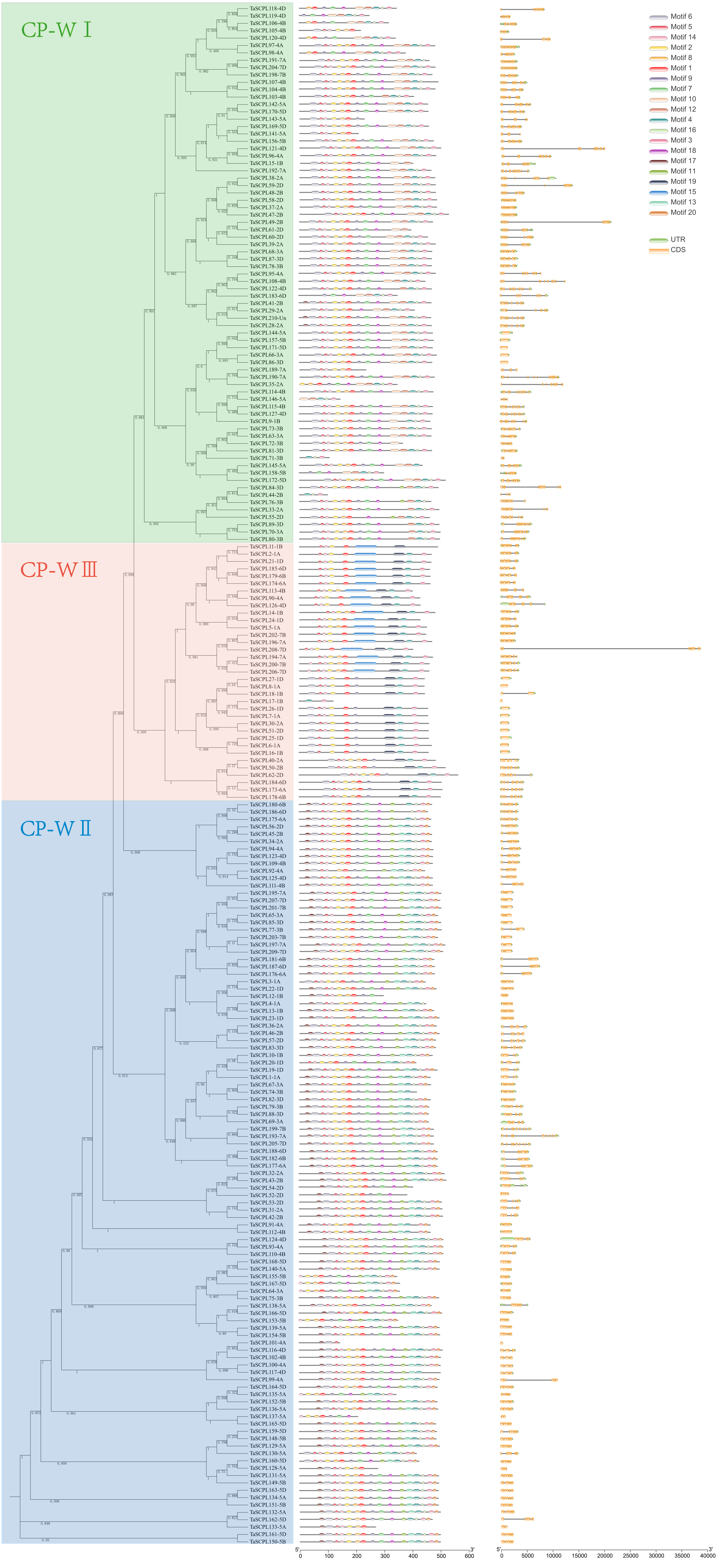

Supplement: Supplementary file 2 — Additional file 2: Figure S2. Phylogenetic relationship, gene structure and conserved motifs analysis of 209 TaSCPL genes. [file 12864_2021_7647_MOESM2_ESM.pdf]
