# Supplementary material for: Genome-wide analysis of the serine carboxypeptidase-like protein family in Triticum aestivum reveals TaSCPL184-6D is involved in abiotic stress response
Source: BMC Genomics. 2021 May 15;22:350. doi: 10.1186/s12864-021-07647-6 (PMC8126144; doi:10.1186/s12864-021-07647-6)
Supplement: Supplementary file 4 — Additional file 4: Figure S4. The overexpression of TaSCPL184-6D increased the germination rate of seeds under PEG6000 treatment. a The phenotypes of WT and TaSCPL184-6D transgenic Arabidopsis seeds under 8 and 10% PEG6000 treatments. b The germination rates of WT and TaSCPL184-6D transgenic Arabidopsis seeds at different time points on MS medium. c The germination rates under 8% PEG6000 treatment. d The germination rates under 10% PEG6000 treatment. A total of three biological replicates were performed. The error bars indicate the standard deviation (SD) of the three replicates. [file 12864_2021_7647_MOESM4_ESM.pdf]

Additional file 4: Figure S4. The overexpression of *TaSCPL184-6D* increased the germination rate of seeds under PEG6000 treatment.

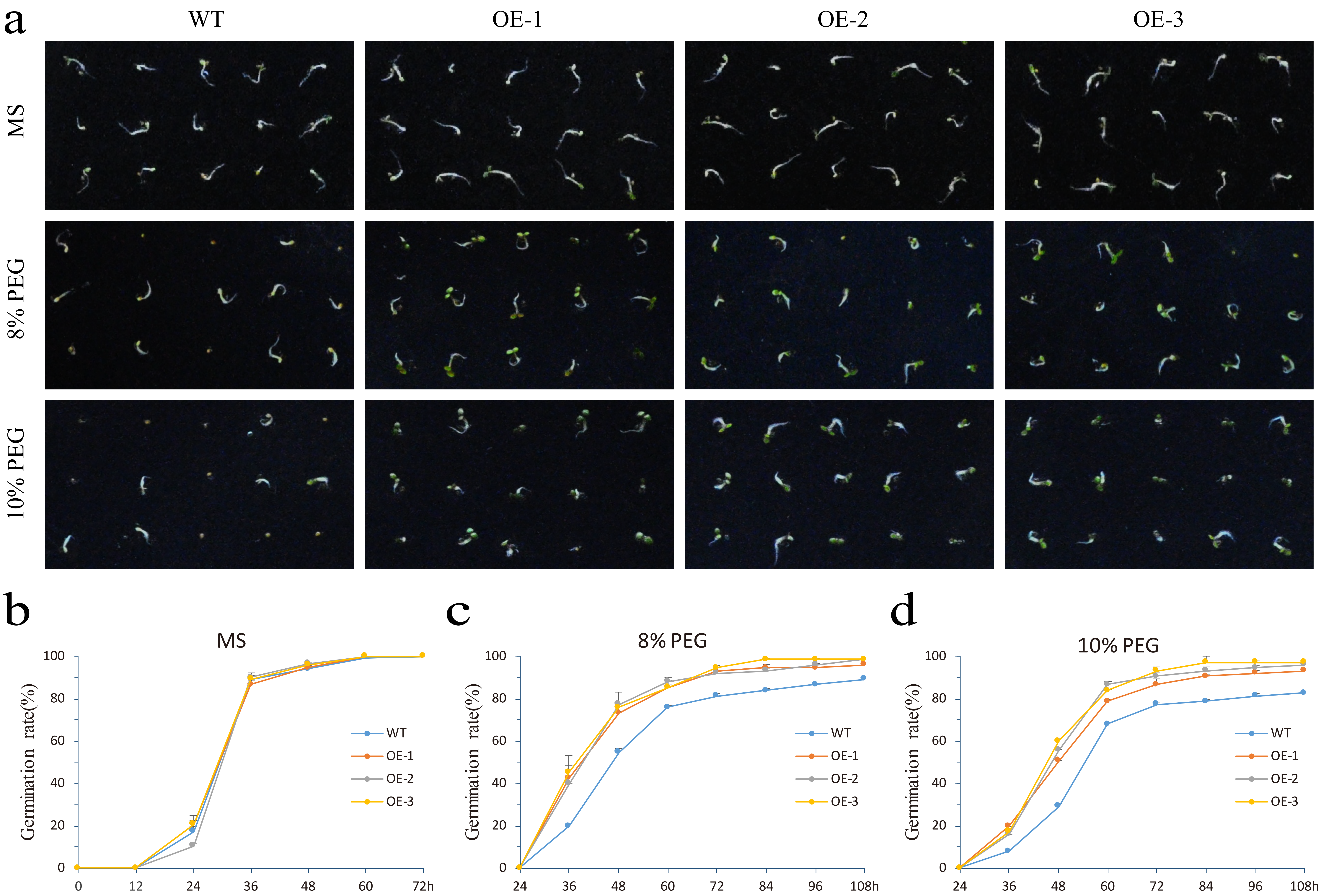

a The phenotypes of WT and *TaSCPL184-6D* transgenic *Arabidopsis* seeds under 8% and 10% PEG6000 treatments. **b** The germination rates of WT and *TaSCPL184-6D* transgenic *Arabidopsis* seeds at different time points on MS medium. **c** The germination rates under 8% PEG6000 treatment. **d** The germination rates under 10% PEG6000 treatment. A total of three biological replicates were performed. The error bars indicate the standard deviation (SD) of the three replicates.
