# Supplementary material for: Genome-wide analysis of the serine carboxypeptidase-like protein family in Triticum aestivum reveals TaSCPL184-6D is involved in abiotic stress response
Source: BMC Genomics. 2021 May 15;22:350. doi: 10.1186/s12864-021-07647-6 (PMC8126144; doi:10.1186/s12864-021-07647-6)
Supplement: Supplementary file 6 — Additional file 6: Figure S6. The overexpression of TaSCPL184-6D enhanced the tolerance to drought and salt stresses in Arabidopsis. a Root length assays of wild-type and TaSCPL184-6D-overexpressing plants on a MS medium. b Root length assays under 10% PEG6000 treatment. c Root length assays under 150 mM NaCl treatment. d Tool root length under 10% PEG6000 treatment. e Tool root length under 150 mM NaCl treatment. f Fresh weight under 10% PEG6000 treatment. g Fresh weight under 150 mM NaCl treatment. [file 12864_2021_7647_MOESM6_ESM.pdf]

Additional file 6: Figure S6. The overexpression of *TaSCPL184-6D* enhanced the tolerance to drought and salt stresses in *Arabidopsis*.

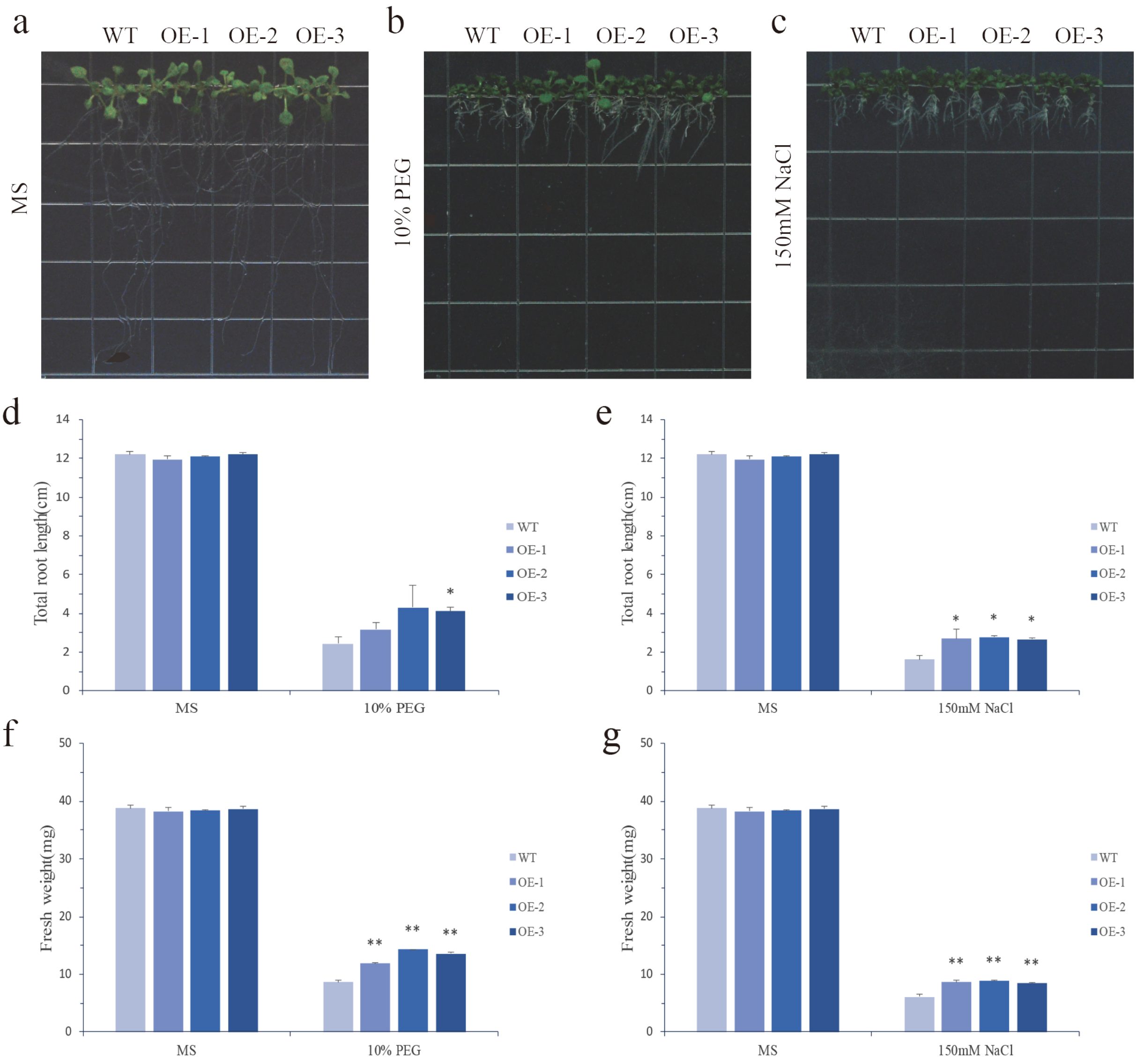

a Root length assays of wild-type and *TaSCPL184-6D*-overexpressing plants on a MS medium. **b** Root length assays under 10% PEG6000 treatment. **c** Root length assays under 150mM NaCl treatment. **d** Tool root length under 10% PEG6000 treatment. **e** Tool root length under 150mM NaCl treatment. **f** Fresh weight under 10% PEG6000 treatment. **g** Fresh weight under 150mM NaCl treatment.
