# Supplementary material for: Genome-wide analysis of the serine carboxypeptidase-like protein family in Triticum aestivum reveals TaSCPL184-6D is involved in abiotic stress response
Source: BMC Genomics. 2021 May 15;22:350. doi: 10.1186/s12864-021-07647-6 (PMC8126144; doi:10.1186/s12864-021-07647-6)
Supplement: Supplementary file 7 — Additional file 7: Figure S7. The overexpression of TaSCPL184-6D enhanced drought tolerance in Arabidopsis. a The drought tolerance phenotypes of WT and TaSCPL184-6D transgenic Arabidopsis in soil. Three-week-old seedlings of WT and TaSCPL184-6D-overexpressing lines were dehydrated for 2 weeks and then rewatered for 3 days. b Statistical analysis of survival rates. c The amount of Pro. d The amount of MDA. Data is shown as the mean ± SD of three independent replicates. Significant differences were observed using a Student’ s t test (*p < 0.05, **p < 0.01). [file 12864_2021_7647_MOESM7_ESM.pdf]

Additional file 7: Figure S7. The overexpression of *TaSCPL184-6D* enhanced drought tolerance in *Arabidopsis*.

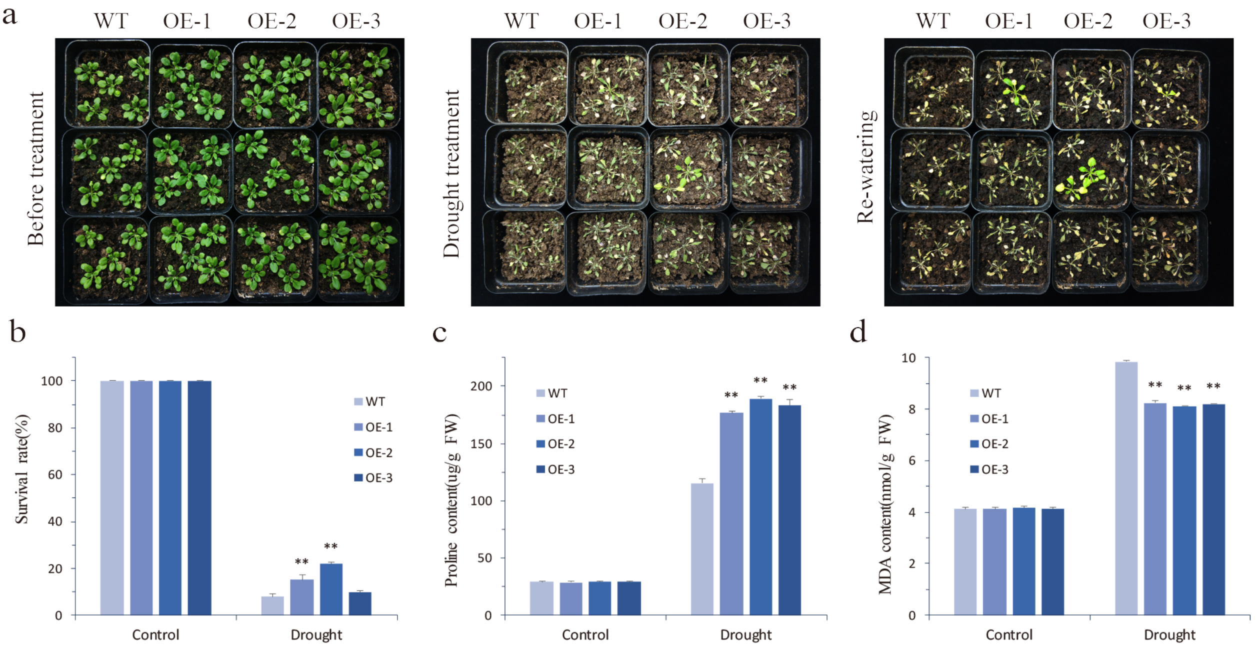

a The drought tolerance phenotypes of WT and *TaSCPL184-6D* transgenic *Arabidopsis* in soil. Three-week-old seedlings of WT and *TaSCPL184-6D*-overexpressing lines were dehydrated for 2 weeks and then rewatered for 7 days. **b** Statistical analysis of survival rates. **c** The amount of Pro. **d** The amount of MDA. Data is shown as the mean ± SD of three independent replicates. Significant differences were observed using a Student' s t test (*$p < 0.05$, **$p < 0.01$).
