# Supplementary material for: Genome-wide analysis of the serine carboxypeptidase-like protein family in Triticum aestivum reveals TaSCPL184-6D is involved in abiotic stress response
Source: BMC Genomics. 2021 May 15;22:350. doi: 10.1186/s12864-021-07647-6 (PMC8126144; doi:10.1186/s12864-021-07647-6)
Supplement: Supplementary file 8 — Additional file 8: Figure S8. The overexpression of TaSCPL184-6D enhanced the tolerance to NaCl tolerance in Arabidopsis. a NaCl tolerance phenotypes of WT and TaSCPL184-6D transgenic Arabidopsis in soil. Three-week-old seedlings of WT and TaSCPL184-6D-overexpressing lines were stressed for 7 days and then rewatered for 3 days. b Statistical analysis of survival rates. c The amount of Pro. d The amount of MDA. [file 12864_2021_7647_MOESM8_ESM.pdf]

Additional file 8: Figure S8. The overexpression of *TaSCPL184-6D* enhanced the tolerance to NaCl tolerance in *Arabidopsis*.

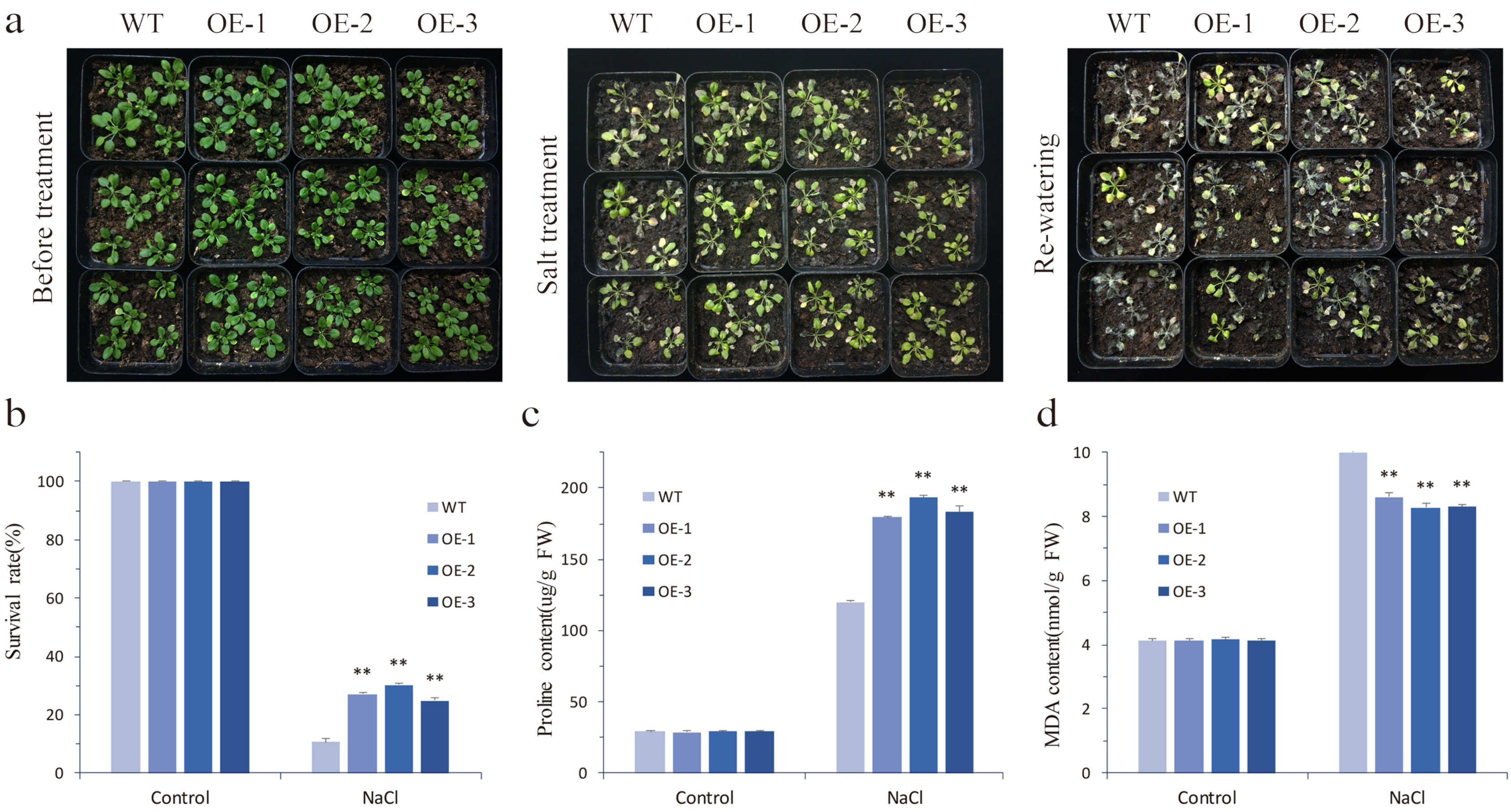

a NaCl tolerance phenotypes of WT and *TaSCPL184-6D* transgenic *Arabidopsis* in soil. Three-week-old seedlings of WT and *TaSCPL184-6D*-overexpressing lines were stressed for 7 days and then rewatered for 7 days. **b** Statistical analysis of survival rates. **c** The amount of Pro. **d** The amount of MDA.
